# The Veterinary Immunological Toolbox: Past, Present, and Future

**DOI:** 10.3389/fimmu.2020.01651

**Published:** 2020-07-28

**Authors:** Gary Entrican, Joan K. Lunney, Sean R. Wattegedera, William Mwangi, Jayne C. Hope, John A. Hammond

**Affiliations:** ^1^The Roslin Institute at The University of Edinburgh, Easter Bush Campus, Midlothian, United Kingdom; ^2^Animal Parasitic Diseases Laboratory, BARC, NEA, ARS, USDA, Beltsville, MD, United States; ^3^Moredun Research Institute, Pentlands Science Park, Edinburgh, United Kingdom; ^4^The Pirbright Institute, Woking, United Kingdom

**Keywords:** immunological toolbox, veterinary, reagents, technologies, databases, monoclonal antibodies

## Abstract

It is well-recognized that research capability in veterinary species is restricted by a lack of immunological reagents relative to the extensive toolboxes for small rodent biomedical model species and humans. This creates a barrier to the strategic development of disease control solutions for livestock, companion animals and wildlife that not only affects animal health but can affect human health by increasing the risk of transmission of zoonotic pathogens. There have been a number of projects aimed at reducing the capability gaps in the veterinary immunological toolbox, the majority of these focusing on livestock species. Various approaches have been taken to veterinary immunological reagent development across the globe and technological advances in molecular biology and protein biochemistry have accelerated toolbox development. While short-term funding initiatives can address specific gaps in capability, they do not account for long-term sustainability of reagents and databases that requires a different funding model. We review the past, present and future of the veterinary immunological toolbox with specific reference to recent developments discussed at the International Union of Immunological Societies (IUIS) Veterinary Immunology Committee (VIC) Immune Toolkit Workshop at the 12th International Veterinary Immunology Symposium (IVIS) in Seattle, USA, 16–19 August 2019. The future availability of these reagents is critical to research for improving animal health, responses to infectious pathogens and vaccine design as well as for important analyses of zoonotic pathogens and the animal /human interface for One Health initiatives.

## Introduction

The development of novel tools and technologies has been fundamental to the advancement of basic and applied immunology across species. The rate of progress of immunological reagent development for veterinary species has been much slower than that for humans and small rodent biomedical model species, and has impacted research capability in those species (1). Historically, however, innovations in surgical procedures in veterinary species have resulted in major step-changes in our understanding of the ontogeny, compartmentalization and function of the immune system. For example, bursectomy in chickens shed new light on mechanisms of B cell development and immunoglobulin production (2), *in utero* thymectomy of lambs revealed the importance of the thymus for lymphocyte development (3) and lymphatic cannulation of sheep revealed that lymphocyte subsets differ between blood, afferent and efferent lymph (4). These ground-breaking experiments were feasible, in part, due to the size of the species under investigation, particularly for the technique of lymphatic cannulation due to the diameter of lymphatic vessels in ruminants (5).

However, this momentum in veterinary immunological studies was not maintained; the vast majority of technological innovations and discoveries in immunology in the past 50 years have been made in mice. The development of congenic mice, differing at a single histocompatibility locus, was a fundamental technological innovation in immunology that led to mice being the primary species of choice for research. That pioneering work of George Snell and the later capability of genetically manipulating congenic mice has allowed immunologists to ascribe functions to genes, molecules and cells with high precision (6). The development of monoclonal antibody (mAb) technology using congenic mice subsequently created almost boundless opportunities for research in basic and translational immunology (7).

The availability of mAbs that could phenotype cells and detect cytokines by ELISA underpinned the discovery of two distinct CD4^+ve^ T-cell subsets in congenic mice (8). The subsequent Th1/Th2 paradigm provided a fundamental framework for investigating immune activation and regulation that has expanded far beyond those original two subsets. Current capability now extends to multi-parametric analyses such as simultaneous fourteen-color flow cytometry that can identify 89 functionally-relevant CD4^+ve^ T-cell subsets in human blood (9). Mass cytometry (CyTOF) methods using panels of well over 40 conjugated antibodies are now allowing for even deeper analysis of single cell expression, offering new insights into cellular subsets and their differentiation (10, 11).

Such technologies cannot usually be applied directly to different species since molecular differences in immunological orthologs result in low cross-reactivity of reagents across species (12) as affirmed by a recent comparison of reactivity of immune protein reagents for other species with swine orthologs (13). Thus, reagent development needs to be evaluated on a case-by-case basis. Gaps in capability for veterinary species are often prioritized based on the extensive mouse and human immunological toolboxes. The expansion of the toolboxes has revealed substantial differences in the ways that humans, mice and veterinary species respond to disease and highlighted to need for studying different species in their own right (14, 15). There have been coordinated efforts to evaluate species cross-reactivity of anti-human CD antigen mAbs through the animal homologs section of the human leukocyte differentiation antigen (HLDA) workshops: for horses (16), dogs (17), pigs (18), and ruminants (19).

In an effort to generate greater international co-ordination for immune reagent characterization activities, the International Union of Immunological Societies (IUIS) Veterinary Immunology Committee (VIC) supported a Toolkit Workshop at the 6th International Veterinary Immunology Symposium (IVIS) in Upsala, Sweden in 2001. This set the scene for a series of VIC Toolkit Workshops (20). It is almost 10 years since the last published review of the veterinary immunology toolbox from the IUIS VIC Toolkit Workshop at the 9th IVIS in Tokyo, Japan (1). Here, we review progress over the past decade by reporting on the IUIS VIC Toolkit Workshop at the 12th IVIS in Seattle, USA in 2019 and take a forward look to the future of the veterinary immunology toolbox.

## The Past

The success of the HLDA workshops was based on good co-ordination, high-quality work and collective effort by the veterinary immunology community, as well as results from past species-specific CD workshops supported by IUIS VIC. Common standards were applied to the distribution and evaluation of anti-human CD reagents being assessed in different laboratories and the collective generated data being reviewed centrally. The outcome was an evidence-based assessment for the activity of species cross-reactive mAbs, with affirmation that only a limited number of mAb directed against human CD antigens actually cross-react with other animal species (21). These results instilled confidence in the performance of those reagents and promoted their uptake by the research community and industry, including companies that market and sell veterinary immunological reagents.

Although the HLDA workshops were primarily focused on evaluation of species cross-reactive antibodies, they played an important role in informing of capability gaps and therefore the prioritization of reagents for future development. A major step-change in the way veterinary immunological reagent development was supported came with the inception of a UK Immunological Toolbox funded by the Biotechnology and Biological Sciences Research Council (BBSRC) and the Scottish Executive Environment and Rural Affairs Department (SEERAD) in 2003. This was unique as it united several laboratories within a single project to take a collective multi-species approach to immunological reagent development. This was followed by the Veterinary Immune Reagent Network (VIRN) funded by United States Department of Agriculture (USDA)/National Institute of Food and Agriculture (NIFA) in the US in 2005. Both projects included the creation of databases listing available veterinary immunological reagents, which will be discussed later. They also expanded the emphasis from mAb anti-CD antigens to expression of immune proteins (cytokines and chemokines) and protein reactive mAbs. The US project included direct collaboration with commercial partners to express these immune proteins. The US and UK projects worked together under a Memorandum of Understanding (MOU) to avoid duplication of effort. This MOU was created in the absence of a mechanism for joint international funding by the respective national agencies. The structure, priorities and achievements of these projects has been published previously (1). A key output from these initiatives was an increased recognition of the importance of coordinated, complementary approaches to reagent prioritization and development. Their success has also been reflected by continued support for reagent development initiatives by funders seeking to build on the significant benefits from their original investments, with the assertion that long-term sustainability is essential.

The funding for veterinary immunology reagent development has changed over the past 10 years, moving from the multi-species models of the UK Immunological Toolbox and US VIRN, to single-species projects. With the exception of ruminants, there is very little species cross-reactivity of veterinary reagents, highlighting that the genes involved in immune responses are amongst the most rapidly evolving in vertebrate genomes (22, 23). However, this does not diminish their potential as disease models. BBSRC and USDA/NIFA have supported reagent development projects for ruminants, swine, horses, aquaculture species and poultry in the past 10 years (Box 1). A barrier to formal international collaboration was lifted in 2013 when USDA/NIFA and BBSRC launched a pilot call to support animal disease research of strategic importance to both the US and UK which included the development of veterinary immunological reagents for agriculturally-relevant animal species. The swine toolkit was a landmark first transatlantic veterinary immunology reagent project funded under this initiative in 2015 (Box 1).

Box 1 **Veterinary immunological reagent and technology projects first funded in the period 2010–2020.**

BMGF: Livestock Antibody Hub: Cattle, swine, poultry (2019–2024):

https://www.pirbright.ac.uk/news/2019/11/bill-melinda-gates-foundation-funds-development-pirbright%E2%80%99s-livestock-antibody-hub. To study cattle, pig and poultry antibody responses at high resolution to expand the understanding of protective immunity in those species and that can also be used as models for a range of human infectious diseases.

USDA/NIFA: Cattle (2019–2022):

https://portal.nifa.usda.gov/web/crisprojectpages/1016686-immune-reagents-for-ruminants-with-primary-focus-on-bovine-specific-reagents.html. To develop, and make commercially available, mAb reagents needed to elucidate cattle immune mechanisms by focusing on CD antigens, cytokines, and chemokines and relevant assays.

USDA/NIFA: Swine (2019–2022):

https://portal.nifa.usda.gov/web/crisprojectpages/1019192-development-of-new-swine-reagents-to-broaden-our-understanding-of-immune-correlates-of-protection-and-microbial-pathogenesis.html. To generate priority reagents for swine immune proteins and pipeline them for marketing. Develop SLA class I tetramers and new assays for important swine immune markers.

USDA/NIFA/BBSRC (US-UK Collaborative): Swine (2015–2019):

https://gtr.ukri.org/projects?ref=BB%2FM028232%2F1

https://portal.nifa.usda.gov/web/crisprojectpages/1005670-us-uk-collaborative-swine-immune-toolkit-development-of-new-immune-reagents-for-swine-health-vaccine-and-disease-studies.html. To develop panels of mAb reactive with swine targets (cytokine, chemokines and their receptors) using conventional and phage-display methods. Use resultant mAbs to develop new assays for swine immunity and make the reagents commercially available.

USDA/NIFA: Horse (2015–2019):

https://portal.nifa.usda.gov/web/crisprojectpages/1005524-equine-immune-reagents-development-of-monoclonal-antibodies-to-improve-the-analysis-of-immunity-in-horses.html. To develop and characterize mAbs for the analysis of horse immunity and distribute these to the scientific community for immunological research.

USDA/NIFA: Aquaculture (2016–2020):

https://portal.nifa.usda.gov/web/crisprojectpages/1009003-collaborative-immune-reagent-network-for-aquacultured-species.html. To develop and provide immunological tools and assays to the aquaculture community to advance health for four fish species: rainbow trout, Atlantic salmon, channel catfish and Nile tilapia.

USDA/NIFA: Poultry (2017–2022):

https://reeis.usda.gov/web/crisprojectpages/1012306-development-of-poultry-immune-reagents.html. To identify chicken immune molecules, particularly cytokines, chemokines and cell surface markers, express them as recombinant proteins, and characterize their function. Develop mAbs to the target molecules and use these for multiplexed detection assays.

BBSRC/SG/BioRad: Cattle and Sheep (2012–2015):

https://bbsrc.ukri.org/research/grants-search/AwardDetails/?FundingReference=BB%2FI019863%2F1. To develop reagents and techniques to enable the investigation of the activation and regulation of the immune systems of cattle and sheep with specific reference to cell-surface molecules, intracellular transcription factors and cytokines that can define phenotypically-distinct macrophage, dendritic cell (DC) and T cell subsets.

USDA/NIFA: US Veterinary Immune Reagent Network (2010–2015):

https://portal.nifa.usda.gov/web/crisprojectpages/0221344-us-veterinary-immune-reagent-network.html. To clone, express, develop mAb reagents specific for ruminants, swine, poultry, equine and aquaculture species, sharing methods across species. Work with commercial partner to market expressed proteins for use by veterinary immunology community.

Although we have focused here on projects funded specifically to develop reagents and supporting technologies, this is not intended to ignore the veterinary immunological reagent development that is conducted within disease-driven projects, networks and within strategic programmes of government research institutes across the globe. The challenge is in capturing the outputs of these diverse activities. The websites of commercial reagent suppliers and peer-reviewed publications are sources of validated information on reagent activity. However, they do not capture everything, a particular gap being the paucity of “negative” data when reagents are found to be non-functional or where repeated attempts fail to generate specific antibodies. These are very valuable data as they can potentially prevent the duplication of wasted effort. The solution lies in community engagement for the sharing of knowledge on reagent availability and performance. Workshops such as those hosted by IUIS VIC Toolkit are a focal point for international information exchange, but they do not have the facility to capture, store and disseminate information at a detailed level. It has been recognized for many years that a major unmet need in veterinary immunology is the lack of centralized, non-commercial, searchable reagent databases (20). The original UK Immunological Toolbox (2003–2009) and the US VIRN (2005–2015) both created lists of reagents but the databases were not sustainable beyond the term of funding. This is not surprising as curation is time-consuming, requiring expert knowledge of immunology and information technology input to create web-based interfaces. This also highlighted the problem of sustainability when there is reliance on short-term funding for reagent development projects. Finding solutions to these problems has been the focus of several recent workshops as discussed below. One exception to this has been the USDA Agricultural Research Service (ARS) supported Porcine Translational Research database (PTRD, http://tinyurl.com/hxxq3ur) (15).

## The Present

The current landscape of the veterinary immunology toolbox has been shaped by new funding approaches to facilitate reagent development while also addressing the complex issues of database construction, collection and validation of data, and sustainability of the database and biobanks of the reagents listed therein. This report summarizes the outcomes of several international workshops where these various elements have been considered.

Before summarizing those outcomes, it is worth reviewing the scope of the toolbox in terms of species coverage and knowledge of immunological capability within those species. In the broadest sense, the concept of a veterinary immunological toolbox encompasses a broad range of livestock, companion animal, biomedical model and wildlife species. There has been progress in reagent development across all of those species in the past 10 years which has been presented at various meetings and workshops. We have identified a number of published articles where reagent availability for different species have been reviewed. For the purposes of the toolbox, livestock species can largely be regarded as belonging to one of four major groupings, namely swine (24, 25), ruminants (22, 26), poultry (27–29), and aquaculture (30, 31). Companion animals include horses (32, 33), cats (34), and dogs (35). As previously discussed, mice are the most common small-animal biomedical model for human (12). However, rabbits (36) and ferrets (37) are also popular small-animal biomedical models for human disease. There is interest in expanding the immunological toolboxes for wildlife species, for example buffalo (38) and badgers (39) due to their potential to act as reservoirs for economically-important livestock diseases. There is also interest in developing immunological reagents for marine mammals such as dolphins (40). In addition, although camelid species are not often regarded as a major target host species for disease studies, they have come to the fore with heightened awareness of MERS-CoV and the potential to reduce zoonotic transmission by investigating vaccine-induced responses in camels (41). Importantly, camels make a unique technological contribution to the immunological toolbox via the production of nanobodies (42, 43).

To date, the concept of the veterinary immunological toolbox has largely (but not exclusively) focused on reagent development for livestock species due to their strategic relevance for funders with a stake in livestock health, food safety and global food security. In the period between the last published review of the IUIS VIC Toolkit Workshop at the 9th IVIS in Tokyo (1) and the IUIS VIC Toolkit Workshop at the 12th IVIS in Seattle, there have been several key meetings whose outcomes are directly relevant to the current status and future directions of the toolbox and merit discussion here. The first was at the 10th IVIS in Milan, Italy in 2013 when BBSRC and The Global Strategic Alliances for the Coordination of Research on the major Infectious Diseases of Animals and Zoonoses (STAR-IDAZ) supported a vaccinology workshop. The lack of immunological tools and reagents was recognized as a major barrier to progress. This can be seen in the subsequent BBSRC Veterinary Vaccinology Strategy (https://bbsrc.ukri.org/about/reviews/scientific-areas/1506-veterinary-vaccinology-strategy/) and the creation of the BBSRC UK Veterinary Vaccinology Network (VVN).

In 2017, BBSRC VVN hosted a workshop to discuss the toolbox initiatives in the UK and US with specific relevance to the aims and objectives of the newly-formed Global Challenges Research Fund (GCRF) International Veterinary Vaccinology Network (IVVN). A full report is available on the BBSRC VVN website (http://www.vetvaccnet.ac.uk/publications/veterinary-immunology-toolbox-meeting-uk-veterinary-vaccinology-network). At this workshop, The Pirbright Institute and The Roslin Institute at the University of Edinburgh announced plans for a new UK Immunological Toolbox project. The combined project would be underpinned by core Institute funding from the BBSRC, with additional support from the BBSRC GCRF Tools and Resources (https://www.immunologicaltoolbox.co.uk/about/funders). This project is addressing major gaps in capability and sustainability. The first of these is the creation of a publicly accessible, searchable database of veterinary immunological reagents to be accessed via a dedicated website. A follow up meeting was held at the VVN Conference in Stirling in early 2018 (https://www.vetvaccnet.ac.uk/news/2018/01/uk-veterinary-vaccinology-network-conference-2018-report) to discuss in more detail the focus of the website and new reagent development. It was agreed by the community that a key driver for the website would be the facility for researchers to submit information on reagent performance and request reagent production where gaps exist. It was discussed that the primary focus of new reagent development should be around T cell and B cell subsets to help dissect in more detail pathogen and vaccine responses. As well as new reagent development the toolbox aims to exploit new technologies to translate current hybridoma stocks into gene blocks via sequencing and create a recombinant antibody pipeline, express recombinant proteins (including cytokines and chemokines), build multiplex platforms and develop high-throughput screening systems for new antibodies. These sequences act as the template from which the constant region can be switched between different species while maintaining target specificity.

A toolkit workshop was held at the 6th European Veterinary Immunology Workshop (EVIW) conference in Utrecht, Netherlands in 2018. Although this conference was organized under the auspices of the European Veterinary Immunology Group (EVIG), as opposed to IUIS VIC, the IUIS VIC Toolkit Committee took a leading role in the organization of the toolkit workshop. Notably, the toolkit workshop was structured to reflect four newly-formed major livestock groupings (swine, ruminants, poultry, aquaculture) of IUIS VIC Toolkit which were announced for the first time at this meeting. The leaders of the species groups represented their respective areas at the workshop. They are listed on the IUIS VIC webpage and can be contacted by members of the community who are seeking information or looking to engage in reagent development for each of those areas (https://iuis.org/committees/vic/). The workshop covered the major projects in Europe and the US on reagent development, including a presentation on the plans for the new UK Immunological Toolbox. In the panel discussion, there was broad international support for the approaches being taken within the new toolbox project and recognition of the complementary work being supported by USDA/NIFA in all of the target species (Box 1). This meeting cemented the requirement for community engagement in the website to provide and maximize information exchange about the availability and performance of reagents and the focus on the generation of novel antibodies and methods to distinguish T and B cell subsets. This particular area will be advanced by the development of a new Livestock Antibody Hub centered at The Pirbright Institute which aims to improve both animal and human health globally by translating research outcomes in livestock diseases (Box 1). A core aim of this Antibody Hub is to develop tools, techniques and reagents for livestock research that bring the research capability to the same level as that for humans and mice.

The IUIS VIC Toolkit workshop at the 12th IVIS in Seattle was the forum for the international launch of the Pirbright/Roslin UK Immunological Toolbox website and the associated database (http://www.immunologicaltoolbox.co.uk). This database was built around the original information collated during the 2003–2009 BBSRC SEERAD-funded UK Immunological Toolbox and is therefore skewed toward three of the four major livestock groupings (swine, ruminants and poultry). However, aquaculture species, companion animals and now major animal pathogens are also included, and as the community engages the amount of information will increase. The main aim of the website is to collate reagent information and act as a centralized source to increase information exchange but is not the only source for any particular species. For example, the USDA Porcine Translational Research Database (http://tinyurl.com/hxxq3ur) is considered a very wide ranging and valuable community resource and cannot be duplicated but information is shared with the UK Immunological Toolbox via mutual awareness and direct communication.

The UK Immunological Toolbox database contains data on reagents that are held in research laboratories, and also those available commercially, which immediately raises questions on the quality and reproducibility of reagents from different sources. The standardized production, evaluation and storage of commercially-available reagents would be expected to reduce batch-to-batch variation, whereas the same reagent produced and stored in different research laboratories is likely to have more variability due to the different conditions. When reagents are listed on the UK Immunological Toolbox website there will be information on their specificity and performance, preferably supported by peer-review publication wherever possible.

There is also a facility for registered users to provide feedback on performance to add to the available information. Such information will be checked before posting against the user's identification. It was emphasized that such a database can be as complete and useful as the community wants it to be. The website and database will be curated centrally, but the community has to take collective ownership by submitting reagents and information on their performance. It is pleasing to see that this is already happening. The toolbox website also serves as a reference point for non-veterinary immunologists looking to expand their choice of biomedical models and facilitate comparative immunology research (44).

Finally, several new opportunities were identified during the open discussion at the IUIS VIC Toolkit Workshop in Seattle. These included the unique opportunity to salvage and store “orphan” mAbs via the sequencing technology within the UK Immunological Toolbox. The preservation of sequences does not incur the high costs associated with maintaining hybridoma cells in liquid nitrogen. In addition, the sharing of sequences circumvents many of the logistical and financial issues involved in the shipment of live cells, particularly across international borders.

## The Future

As we enter the third decade of the 21st century, the “One Health” agenda has never been more important. The development of solutions for controlling infectious diseases in livestock, companion animals and wildlife not only has direct benefits for the target species but can reduce disease transmission across species, including zoonotic transmission, thereby reducing the wider global disease burden (45). Close contact between different animal species and between animals and humans is a risk for zoonotic disease, which can be difficult to manage in low and middle income countries (LMICs) (46). Given the importance of livestock to LMICs, the veterinary immunological toolbox provides economic and health benefits by underpinning animal vaccine development.

The quality of toolbox reagents and associated information in the UK Immunological Toolbox database are paramount. Evidence-based validation and standardization of new technologies is essential to generate confidence in performance and encourage uptake by the community. There remain major capability gaps in multi-analyte protein technologies for veterinary species. The development of such technologies is technically challenging, but entirely feasible with the appropriate resources and effort. The key to success is in working together. The single-tube technology that simultaneously identifies 89 functionally-relevant CD4^+ve^ T-cell subsets in human blood was developed and validated through the collective efforts of the multiple partners in the EuroFlow and PERISCOPE consortia (9). Multiparametric technologies are extremely powerful; one way of expanding the flexibility of the relatively limited range of antibodies in veterinary species is the ability to efficiently conjugate small amounts of antibodies with different labels for defining immune correlates. The identification and quantification of immunological correlates of protection are aspirational goals for the development of safe and effective vaccines (47, 48). However, with the exception of anti-virus neutralizing antibodies, immunological correlates of protection tend to be multifactorial rather than singular, particularly in the case of cell-mediated protective immunity requiring not only cell subset identification but appropriate cytokine co-expression. The solution to identifying such correlates lies in the application of a range of multi-plex technologies that all detect multiple analytes at the genetic, protein, and cellular level, so called “systems vaccinology” (49).

We are also moving into an era of high dependency on computational infrastructure as the data generated by such complex studies require specialized programmes for full analysis. Hence, collective approaches are becoming increasingly important if we are to maximize our potential to develop and adopt complex technologies in the future. The importance of genomic information and alternate expression systems such as *Pichia pastoris*, insect and mammalian cells has meant wider availability of species-specific immune proteins. The veterinary immunology community has a long history of working together for collective good, such as the HLDA workshops, international CD workshops, toolkit committees, collaborative funding initiatives and the immunological toolbox. In doing so we need to maintain a global perspective and consider technologies that create solutions for animal diseases across borders. One example is the antibody sequencing technologies of the new UK Immunological Toolbox. In addition to the advantages described earlier, this technology offers particular cost-effective and sustainability benefits for the transfer and storage of reagents to LMICs where veterinary immunology research is being conducted.

In parallel to sequencing, expressing and engineering mAbs, companies and research groups all over the world are adapting single B cell sequencing technologies to a range of host species. These technologies often rely to some extent on existing reagents to identify B cells (including antigen specific B cells) but are generally very adaptable to any given species and synergise well with existing mouse recombinant antibody expression methods. These methods are providing a completely new route to identifying antibodies against specific epitopes on pathogens as well as other foreign immunizing antigens. These antibodies can be used as reagents, including mapping complex epitope landscapes to inform structural vaccinology approaches to increase efficacy, and may also be used as therapeutics. Antibodies are now a primary therapeutic goal of many companies for a range of human diseases. Cats and dogs are not only a profitable target market for immunotherapeutics, they provide value data on *in vivo* mAb function (50). Although the cost of such treatments is currently prohibitive for food producing species, large animal models and species-specific reagents can have a very important role in testing manufacture, delivery and efficacy of mAbs as part of the One Health approach.

The impact of veterinary immunology research will ultimately be measured by the development, or contribution to the development, of disease-control solutions including diagnostic tests, vaccines and genetic-based strategies. The range of vaccine-delivery platforms is rapidly expanding, including improved adjuvants, vector-based delivery systems and genetic vaccination with DNA and RNA. Although viral-vectored vaccines are successfully deployed in humans and companion animals (51), public safety concerns remain regarding their use food animals (52). The immunological toolbox can be applied to safety and efficacy studies in livestock, thereby informing on the benefit-risk ratio that would be impossible to do at the same scale in humans or primates.

Animal genetics can provide insights into responses to infection and vaccination which can be translated into livestock breeding programmes (53, 54). Breeding programmes require several generations to observe population effects and conclusive proof for the effect of a specific genotype on immune status requires functional evidence, hence reliance on the toolbox. New gene-editing technologies such as CRISPR now allow very targeted approaches to livestock production (55). This is the future of livestock farming and the immunological toolbox not only has a role to play in the identification of genes to be targeted, but it will also be important for defining subsequent immune function, including potential off-target effects. Genome editing is also creating the opportunities to engineer species to act as better models for human diseases alongside or in addition to genetically defined and tailored breeds, such as SCID pigs and MHC homozygous pigs (56, 57). For example, pigs are emerging as a very powerful model to predict human influenza vaccine responses but to achieve the maximum benefit of such models a complete toolkit is required (58). Gene editing is already providing pig organs for future human xenotransplantation, a biomedical application that has helped drive reagent development in pigs (59, 60).

## Conclusion

The veterinary immunological toolbox is very broad in its scope and has evolved from multiple efforts across the globe. In the broadest sense, the toolbox incorporates livestock, companion animals, wildlife and biomedical animal species. Each is important in its own right, but all are collectively important for the One Health agenda and for controlling existing and emerging diseases that infect different animal populations and have zoonotic potential. As human populations expand, there is a need to protect food security without compromising food safety. Disease prevention and control results in improvements in animal health and welfare, which not only has economic and ethical benefits but can also address concerns for climate change by making food production more efficient. Basic immunology underpins these approaches, from vaccine design to understanding the effects of gene editing. The immunological toolbox website and associated searchable database provides a new focal point for information and knowledge exchange for the veterinary immunology community. The key to future success is global collective working facilitated by networks such as national immunological societies, EVIW, IVVN, American Association of Veterinary Immunologists (AAVI), and IUIS VIC Toolkit Committee.

## Author Contributions

All authors listed have made a substantial, direct and intellectual contribution to the work, and approved it for publication.

## Conflict of Interest

The authors declare that the research was conducted in the absence of any commercial or financial relationships that could be construed as a potential conflict of interest.

## Abbreviations

BMGF: Bill & Melinda Gates Foundation
BBSRC: Biotechnology and Biological Sciences Research Council
NIFA: National Institute of Food and Agriculture
REEIS: Research, Education and Economics Information System
SG: Scottish Government
USDA: United States Department of Agriculture.
